# First-Line Pembrolizumab Monotherapy for Advanced Non-Small Cell Lung Cancer: A Multicenter Real-World Study from Vietnam

**DOI:** 10.3390/curroncol33040215

**Published:** 2026-04-14

**Authors:** Thi Huong Pham, Cam Phuong Pham, Thi Thu Huong Nguyen, Khanh Toan Nguyen

**Affiliations:** 1 Department of Oncology, Hanoi Medical University, Hanoi 100000, Vietnam; bshuongn2ub@gmail.com (T.H.P.); nguyenthu_huong@hmu.edu.vn (T.T.H.N.); 2 Nghe An Oncology Hospital, Nghe An 43000, Vietnam; 3 Nuclear Medicine and Oncology Center, Bach Mai Hospital, Hanoi 100000, Vietnam; phamcamphuong@hmu.edu.vn; 4 Nuclear Medicine Department, Hanoi Medical University, Hanoi 100000, Vietnam; 5 Oncology and Nuclear Medicine Department, University of Medicine and Pharmacy, Vietnam National University, Hanoi 100000, Vietnam; 6 Vietnam National Cancer Hospital, Hanoi 100000, Vietnam

**Keywords:** non-small cell lung cancer, advanced stage, pembrolizumab monotherapy, Vietnam

## Abstract

Background: This study aimed to evaluate the effectiveness and safety of first-line pembrolizumab monotherapy in patients with advanced non-small cell lung cancer (NSCLC) in real-world clinical practice in Vietnam. Methods: We performed a multicenter retrospective cohort study of patients with locally advanced or metastatic NSCLC who received first-line pembrolizumab monotherapy in Vietnam between January 2018 and August 2024. The primary endpoints were progression-free survival (PFS), overall survival (OS), and safety profile. Results: A total of 73 patients were included, with a median age of 69 years (range, 47–92). Most patients had good performance status (ECOG PS 0–1, 75.3%) and high PD-L1 expression (TPS ≥ 50%, 86.3%). The overall response rate was 60.3%, and the disease control rate was 79.5%. Median PFS was 11.3 months (95% CI, 6.9–15.8), and median OS was 25.4 months (95% CI, 20.8–30.0). Multivariate analysis identified never-smoking status (HR 3.14, 95% CI 1.16–8.50; *p* = 0.024), squamous histology (HR 4.09, 95% CI 1.18–14.17; *p* = 0.026), and low PD-L1 expression (TPS 1–49%) (HR 3.67, 95% CI 1.14–11.78; *p* = 0.029) as independent predictors of inferior overall survival. Immune-related adverse events, including pneumonitis, hepatitis, nephritis, fever, skin reactions, and myositis, were mostly mild and manageable, with grade 3 toxicity occurring in only 4.2% of patients. Better survival was observed in patients with high PD-L1 expression and non-squamous histology. However, the association with non-squamous histology should be interpreted with caution due to the very small number of squamous cases. Conclusions: First-line pembrolizumab monotherapy demonstrated favorable effectiveness and acceptable safety in patients with advanced NSCLC in real-world clinical practice in Vietnam. Clinical outcomes were particularly favorable in patients with high PD-L1 expression, non-squamous histology, and a history of smoking. Nevertheless, the survival benefit associated with non-squamous histology should be interpreted cautiously, given the limited number of patients with squamous histology. These findings support the use of pembrolizumab monotherapy in selected patient populations within resource-limited settings.

## Simple Summary

Lung cancer is one of the most common malignancies and the leading cause of cancer-related death in Vietnam. Pembrolizumab monotherapy is approved as a first-line treatment for patients with advanced non-small cell lung cancer (NSCLC) without *EGFR* or *ALK* mutations and with PD-L1 expression ≥1%. In this multicenter, real-world study from Vietnam, 73 patients with advanced NSCLC received first-line pembrolizumab monotherapy and achieved favorable survival outcomes consistent with those reported in other real-world studies. Better results were observed in patients with high PD-L1 expression, non-squamous histology, and a history of smoking. Adverse events were mostly mild and manageable. However, the survival benefit associated with non-squamous histology should be interpreted with caution due to the very small number of patients with squamous histology. These findings support the use of pembrolizumab monotherapy in carefully selected patients with advanced NSCLC, even in resource-limited settings.

## 1. Introduction

Lung cancer remains the most frequently diagnosed malignancy and the leading cause of cancer-related mortality worldwide. According to the GLOBOCAN 2022 database, lung cancer accounts for approximately 12.4% of all newly diagnosed cancers and 18.7% of total cancer deaths globally [1]. In Vietnam, lung cancer represents a substantial public health burden, with an estimated 24,426 new cases and 22,597 deaths annually, ranking second in cancer-related mortality. Lung cancer has a poor prognosis due to its aggressive biological invasiveness, high propensity for metastasis, and prolonged asymptomatic latent period. Therefore, most patients present with metastases at diagnosis. According to the Surveillance, Epidemiology, and End Results (SEER) program—the U.S. National Cancer Institute’s national registry—the 5-year relative survival rate for lung cancer is 28.1% [2]. In Vietnam, this rate is lower, approximately 15% [3,4].

Non-small cell lung cancer (NSCLC) constitutes approximately 85% of all lung cancer cases. Historically, treatment options for patients with advanced or metastatic NSCLC lacking actionable driver mutations were limited to platinum-based cytotoxic chemotherapy, which conferred modest survival benefits, with a median OS of only 6–8 months and substantial treatment-related toxicities [5]. The introduction of immune checkpoint inhibitors (ICIs) targeting the programmed cell death-1 (PD-1) receptor and its ligand (PD-L1) has dramatically transformed the therapeutic landscape of NSCLC, resulting in significant improvements in survival outcomes and quality of life [6,7].

In the KEYNOTE-024 trial, pembrolizumab significantly improved progression-free survival (median 10.3 months) and overall survival (median 26.3 months) with a more favorable toxicity profile and better quality of life compared with platinum-based chemotherapy in patients with advanced NSCLC, high PD-L1 tumor proportion score (TPS ≥ 50%), and no *EGFR* or *ALK* genomic alterations. Consequently, in 2016, the U.S. Food and Drug Administration (FDA) approved pembrolizumab as first-line monotherapy for this patient population [8,9,10]. Subsequent studies, including KEYNOTE-042, extended the indication to patients with PD-L1 TPS ≥ 1%, although the magnitude of clinical benefit was more pronounced among those with high PD-L1 expression [11,12].

Despite the robust evidence from RCTs, the strict eligibility criteria and controlled settings of these trials often limit their generalizability to routine clinical practice. Real-world populations frequently include older patients, those with poorer performance status, multiple comorbidities, and heterogeneous disease characteristics, who are underrepresented in clinical trials. Consequently, real-world evidence (RWE) studies are essential to complement RCT data by providing insights into treatment effectiveness, safety, and prognostic factors in broader and more diverse patient populations.

In Vietnam, lung cancer continues to be a major public health challenge, with one of the highest smoking prevalence rates in Southeast Asia, particularly among men [1].

Pembrolizumab has been approved for the treatment of advanced NSCLC since 2017.

Despite recent advances in immunotherapy, access to these treatments is still limited due to significant financial constraints and restricted health insurance coverage. To date, there is a paucity of published real-world data evaluating the outcomes of pembrolizumab monotherapy in Vietnamese patients with advanced NSCLC. Therefore, we conducted a multicenter real-world study to assess the effectiveness, safety, and prognostic factors associated with first-line pembrolizumab monotherapy in patients with advanced NSCLC in Vietnam. Our findings aim to provide clinically relevant evidence to guide treatment decisions in resource-limited settings.

## 2. Methods

### 2.1. Study Design and Setting

This was a multicenter, retrospective observational cohort study conducted across five major oncology centers in Vietnam, including the National Cancer Hospital (K Hospital), Bach Mai Hospital, Hanoi Medical University Hospital, National Lung Hospital, and Nghe An Oncology Hospital. Patients were enrolled between January 2018 and August 2024.

### 2.2. Study Population

#### 2.2.1. Inclusion Criteria

Eligible patients met all of the following criteria:

1. Age ≥ 18 years.
2. Histologically or cytologically confirmed diagnosis of NSCLC.
3. Locally advanced disease (stage IIIB–IIIC) not suitable for definitive concurrent chemoradiotherapy, or metastatic disease (stage IV), according to the 8th edition of the American Joint Committee on Cancer (AJCC) TNM classification; or documented disease recurrence/progression at least 6 months after completion of curative-intent therapy.
4. Eastern Cooperative Oncology Group (ECOG) performance status (PS) of 0–2.
5. Tumor PD-L1 expression tumor proportion score (TPS) ≥ 1%, assessed by immunohistochemistry.
6. Absence of sensitizing *EGFR* mutations or *ALK* rearrangements.
7. No prior systemic therapy for advanced disease.

#### 2.2.2. Exclusion Criteria

Patients were excluded if they had:

1. A history of another active malignancy.
2. Active autoimmune disease requiring systemic treatment within the previous two years.
3. Active interstitial lung disease or pneumonitis requiring systemic corticosteroid therapy.

### 2.3. Data Collection

Clinical data were retrospectively collected from electronic medical records and institutional databases, including:

- Demographic characteristics: age, sex, smoking history.
- Clinical variables: ECOG performance status, comorbidities (including chronic obstructive pulmonary disease [COPD]), disease stage, and presence of brain metastases.
- Pathological and molecular features: histological subtype, PD-L1 TPS, *EGFR*, *ALK*, and *KRAS* mutation status.
- Treatment characteristics: number of pembrolizumab cycles, local therapies (radiotherapy or surgery), treatment discontinuation, and subsequent therapies.

The lung immune prognostic score (LIPS-3), incorporating ECOG PS, neutrophil-to-lymphocyte ratio (NLR), and baseline corticosteroid use, was calculated for each patient [13].

Follow-up time was calculated from the date of pembrolizumab initiation to the date of death, disease progression, or last follow-up, whichever occurred first. The median follow-up time was estimated using the reverse Kaplan–Meier method. Patients lost to follow-up or alive at the cutoff date were censored in survival analyses.

### 2.4. Molecular and Pathological Assessment

PD-L1 expression was assessed using the PD-L1 IHC 22C3 pharmDx assay (Dako, Agilent Technologies, Santa Clara, CA, USA) and reported as tumor proportion score (TPS). *EGFR* mutations were primarily detected by polymerase chain reaction (PCR) using the AmoyDx EGFR 29 Mutation Detection Kit (Amoy Diagnostics, Haicang, Xiamen, China) or by next-generation sequencing (NGS) on the NextSeq platform (Illumina, San Diego, CA, USA). *ALK* rearrangements were identified using immunohistochemistry, PCR, or NGS. *KRAS* mutations and other genomic alterations were analyzed using NGS or PCR.

### 2.5. Treatment Protocol

All patients received pembrolizumab at a fixed dose of 200 mg administered intravenously every 3 weeks (Keytruda^®^, Merck & Co., Inc., Kenilworth, NJ, USA). Treatment was continued for up to 35 cycles or until disease progression, unacceptable toxicity, withdrawal of consent, or death.

Tumor response was evaluated every three cycles or earlier if clinically indicated, using contrast-enhanced computed tomography or magnetic resonance imaging. Treatment response was assessed according to the Response Evaluation Criteria in Solid Tumors (RECIST), version 1.1.

### 2.6. Study Endpoints

Survival outcomes analyzed in this study were progression-free survival (PFS), overall survival (OS), and safety. PFS was defined as the time from the initiation of pembrolizumab until documented disease progression or death from any cause. OS was defined as the time from treatment initiation until death from any cause. Tumor response was evaluated using RECIST 1.1 criteria, with the objective response rate (ORR) representing the proportion of patients who achieved complete or partial response. All adverse events were systematically graded according to the Common Terminology Criteria for Adverse Events (CTCAE) version 5.0.

### 2.7. Statistical Analysis

Statistical analyses were performed using IBM SPSS Statistics version 25.0 (IBM Corporation, Armonk, NY, USA). Patient demographics, clinical characteristics, and safety data were summarized using descriptive statistics. Survival curves for PFS and OS were estimated using the Kaplan–Meier method and compared using the log-rank test. Multivariate analyses were conducted using Cox proportional hazards regression models to identify independent prognostic factors for survival outcomes. Covariates included age (<70 vs. ≥70), sex, ECOG PS (0–1 vs. 2), smoking status, COPD, histology (squamous vs. non-squamous), LIPS-3 score (favorable vs. unfavorable), presence of brain metastases, PD-L1 TPS (≥50% vs. 1–49%), and *KRAS* mutation status. A two-sided *p*-value < 0.05 was considered statistically significant.

## 3. Results

### 3.1. Patient Characteristics

A total of 73 patients with advanced NSCLC treated with first-line pembrolizumab monotherapy were included in this analysis. The median age was 69 years (range 47–92), and 91.8% of the patients were male. The majority of patients (75.3%) had a good performance status (ECOG PS 0–1), while 24.7% had an ECOG PS of 2. Regarding smoking status, 42.5% were current smokers and 31.5% were former smokers.

At diagnosis, 87.7% of patients had stage IV disease, including 16.4% with brain metastases. Adenocarcinoma was the predominant histological subtype (83.6%), followed by squamous cell carcinoma (9.6%) and other histologies (6.8%). Most patients exhibited high tumor PD-L1 expression, with 86.3% having TPS ≥ 50% and 13.7% having TPS of 1–49%. Additional baseline characteristics are summarized in Table 1.

### 3.2. Treatment Exposure and Follow-Up

At the data cutoff date of 31 December 2024, the median follow-up duration was 18.6 months (95% CI: 13.1–24.0 months). Overall, 45 of 73 patients (61.6%) had experienced disease progression or death. The mean number of pembrolizumab cycles administered was 10.7 (range, 2–35).

### 3.3. Treatment Response 

The overall response rate (ORR) of the study was 60.3%, with no patients achieving a complete response (CR). The stable disease (SD) rate was 19.2%, and 15 patients (20.5%) experienced progressive disease (PD). The disease control rate (DCR) reached 79.5% (Table 2).

### 3.4. Survival Outcomes

**Figure 1:**
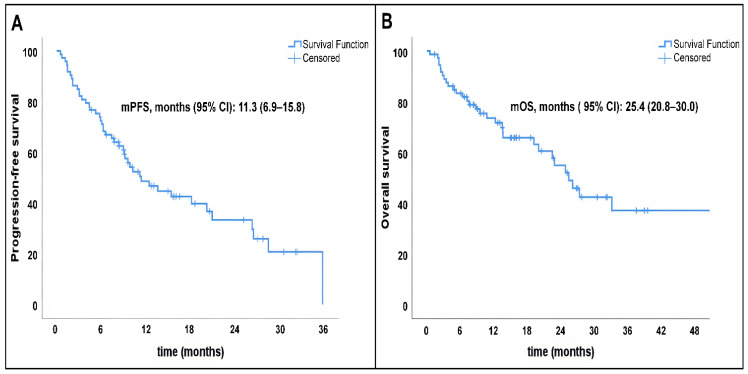
(**A**) Progression-free survival. (**B**) Overall survival.

The median progression-free survival (PFS) was 11.3 months (95% CI, 6.9–15.8), as illustrated in Figure 1A. The median overall survival (OS) was 25.4 months (95% CI, 20.8–30.0), with 1-year and 2-year OS rates of 71.7% and 52.0%, respectively (Figure 1B).

**Figure 2:**
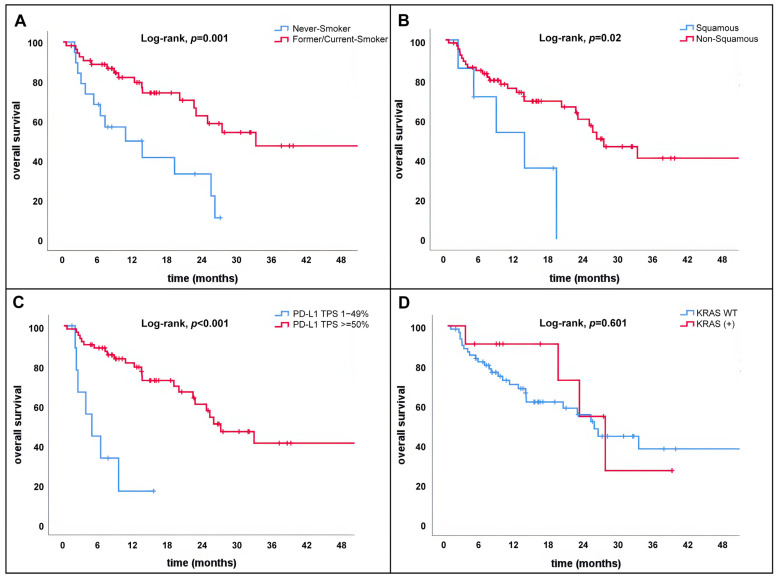
(**A**) OS of patients according to smoking status. (**B**) OS of patients according to histopathology. (**C**) OS according to PD-L1 expression tumor proportion score. (**D**) OS of patients according to *KRAS* gene mutation status.

In subgroup analyses, patients younger than 70 years demonstrated significantly longer OS compared with those aged ≥ 70 years (33.1 vs. 24.8 months; *p* = 0.032). Patients with good performance status (ECOG PS 0–1) had superior OS compared with those with PS 2 (27.3 vs. 6.4 months; *p* = 0.002). Current or former smokers achieved significantly longer OS than never-smokers (33.1 vs. 10.7 months; *p* = 0.001) (Figure 2A).

Furthermore, patients with non-squamous histology, favorable LIPS-3 scores, and high PD-L1 expression (TPS ≥ 50%) experienced significantly better OS than their respective counterparts. Specifically, median OS was 27.3 months for non-squamous tumors versus 13.6 months for squamous tumors (*p* < 0.05), 27.3 months for favorable LIPS-3 versus 19.1 months for unfavorable scores (*p* < 0.05), and 27.3 months for PD-L1 TPS ≥ 50% versus 4.8 months for TPS 1–49% (*p* < 0.001). Kaplan–Meier survival curves for these subgroup analyses are presented in Figure 2B,C.

### 3.5. Prognostic Factors for Overall Survival

Multivariate Cox proportional hazards regression analysis identified never-smoking status (hazard ratio [HR] 3.14; 95% CI, 1.16–8.50; *p* = 0.024), squamous histology (HR 4.09; 95% CI, 1.18–14.17; *p* = 0.026), and low PD-L1 expression (TPS 1–49%) (HR 3.67; 95% CI, 1.14–11.78; *p* = 0.029) as independent predictors of inferior OS (Table 3).

Other clinical variables, including age, sex, ECOG performance status, LIPS-3 scores, COPD, presence of brain metastases, and *KRAS* mutation status, were not independently associated with OS.

### 3.6. Safety

The safety profile of pembrolizumab is summarized in Table 4. The most frequent hematologic adverse event was anemia, observed in 46.6% of patients, predominantly grade 1–2, with only two cases (2.8%) of grade 3–4 anemia. Grade 3 neutropenia occurred in one patient (1.4%), and grade 4 thrombocytopenia was reported in one patient (1.4%). Any-grade hepatotoxicity was observed in 58.9% of patients. Immune-related adverse events (irAEs) included pneumonitis (8.2%), skin reactions (15.1%), fever (9.6%), myositis (2.8%), hepatitis (1.4%), diarrhea (1.4%), and nephritis (4.1%). Most irAEs were mild (grade 1–2) and manageable. Grade 3 hepatitis occurred in 1.4% of patients and resulted in treatment delays. Only one patient (1.4%) developed grade 3 pneumonitis, which led to permanent treatment discontinuation. No treatment-related deaths were observed.

## 4. Discussion

To our knowledge, this study represents the first and largest multicenter real-world investigation evaluating the effectiveness and safety of first-line pembrolizumab monotherapy in patients with advanced non-small cell lung cancer (NSCLC) in Vietnam. Most patients had PD-L1 TPS ≥ 50%, while 13.7% had PD-L1 TPS 1–49%. Of these, 10 patients (13.7%) received pembrolizumab monotherapy, primarily due to poor performance status (ECOG PS ≥ 2), multiple comorbidities (e.g., hypertension, diabetes mellitus, cardiovascular disease, and chronic obstructive pulmonary disease…), and advanced age. Financial constraints also influenced treatment decisions in some cases. In a cohort reflecting routine clinical practice, pembrolizumab demonstrated favorable clinical outcomes, with a median progression-free survival (PFS) of 11.3 months and a median overall survival (OS) of 25.4 months, alongside an acceptable safety profile.

### 4.1. Comparison with Pivotal Clinical Trials and Real-World Studies

The median PFS observed in our study (11.3 months) is slightly longer than that reported in the pivotal KEYNOTE-024 trial (10.3 months). Our findings are also consistent with real-world studies from France, where median real-world PFS ranged between 10.1 and 11.5 months [14,15,16]. Similarly, a multicenter retrospective study in Europe of patients with NSCLC and PD-L1 TPS ≥ 50% treated with pembrolizumab monotherapy reported a median time on treatment (ToT) of 14 months (95% CI: 10.5–17.5 months) [17]. In the large international real-world Pembro-real 5Y study (2025), which included 1050 patients treated with first-line pembrolizumab monotherapy across 61 institutions in 14 countries (median follow-up of 70.3 months), the real-world progression-free survival (rw-PFS) was 10.4 months, the 5-year overall survival rate was 26.9% (95% CI 23.8–30.2%), and the median overall survival (OS) was 21.8 months (95% CI 19.1–25.7) [18]. Conversely, somewhat shorter PFS values have been reported in cohorts from Spain, Italy, Japan, and China, with a median PFS of approximately 7.5–10 months [19,20,21,22,23].

Notably, the KEYNOTE-042 trial, which extended pembrolizumab monotherapy to patients with PD-L1 TPS ≥ 1%, reported a median PFS of only 7.1 months among patients with TPS ≥ 50%, substantially inferior to KEYNOTE-024. In the KEYNOTE-042 trial, 53.1% of patients had low PD-L1 expression (TPS 1–49%). The authors did not offer any specific explanation for the observed difference in PFS, apart from highlighting the heterogeneity of the study population. Several factors may explain the differences in outcomes between our study and the KEYNOTE-024 trial. First, our cohort had a high proportion of non-squamous histology (90.4%), which is associated with a better prognosis. Second, unlike KEYNOTE-024, which included only metastatic patients, our study also enrolled patients with locally advanced unresectable disease or post-curative relapse. Third, brain metastases were present in 16.4% (12/73) of our patients, most of whom received combined local therapy with Gamma Knife radiosurgery and whole-brain radiotherapy. Additionally, differences in access to supportive care services and the intensity of follow-up schedules may have also influenced treatment outcomes.

In addition, real-world studies from East Asia, particularly Japan, reported lower PFS, likely due to older patient age, poorer performance status (ECOG PS ≥ 2 in ~30% of cases), and higher rates of baseline brain metastases.

Our median OS of 25.4 months closely approximates that reported in KEYNOTE-024 (26.3 months). A systematic review of 41 real-world evidence (RWE) studies encompassing more than 7600 previously untreated patients with advanced NSCLC receiving pembrolizumab monotherapy reported considerable variability in survival outcomes, with median OS ranging from 3.0 to 34.6 months [24]. Although RWE studies reported a median OS shorter than that in the KEYNOTE-024 trial, approximately half of the reported median OS values fell within the 95% confidence interval (CI) of the OS reported in KEYNOTE-024 (18.3–40.4 months). Patients with disease stage and performance status characteristics similar to those in the KEYNOTE-024 trial derived comparable benefit from pembrolizumab monotherapy, with median OS ranging from 18.9 to 22.8 months [24]. Importantly, the 1-year and 2-year OS rates of 71.7% and 52.0%, respectively, underscore the durable benefit of pembrolizumab monotherapy in routine practice, even within a resource-limited healthcare setting.

The overall response rate (ORR) in our study reached 60.3%, with a disease control rate (DCR) of 79.5%. Our results are higher than those reported in the KEYNOTE-024 study but remain fairly comparable to other research. According to the PEMBREIZH study by Karim Amrane et al. (2020), the ORR was 57.3% (including a complete response (CR) rate of 2.7%), and the DCR was 71% [14]. Similarly, the DCR reported by Chen Y (2021) in Shanghai, China, reached 87.7% [21]. With a DCR of nearly 80%, patients still derive clinical benefits in terms of quality of life, even when a CR is not achieved.

### 4.2. Prognostic Factors and Clinical Implications

We observed that in univariate analysis, factors such as age < 70 years, smoking history, good performance status (ECOG PS 0–1), favorable LIPS-3 score (0), non-squamous histology, and high PD-L1 expression were associated with better overall survival compared with age ≥ 70 years, never-smoking status, poor performance status (PS 2), unfavorable LIPS-3 score (1–3), squamous histology, and low PD-L1 expression (*p* < 0.05). However, in multivariable analysis using the Cox proportional hazards regression model, never-smoking status, squamous histology, and low PD-L1 expression (1–49%) emerged as independent prognostic factors predictive of increased risk of mortality. The remaining factors did not retain independent prognostic significance for overall survival. These findings provide important insights into patient selection and therapeutic stratification.

PD-L1 expression remains the most clinically validated biomarker for predicting response to pembrolizumab monotherapy. In our cohort, patients with PD-L1 TPS ≥ 50% achieved markedly superior OS compared with those with TPS 1–49% (27.3 vs. 4.8 months), and low PD-L1 expression independently predicted worse survival in multivariate analysis. A meta-analysis conducted in China included 5 randomized controlled clinical trials (RCTs) involving 2877 patients. Age, sex, smoking history, and PD-L1 expression status were used to predict the clinical benefit of pembrolizumab. In patients with TPS < 1%, first-line combination therapy provided an OS benefit, with no relevant data available for monotherapy. In patients with TPS 1–49%, the chemotherapy plus pembrolizumab regimen had an HR 0.60 (95% CI: 0.44–0.81, *p* = 0.0007). Only one study addressed monotherapy, with an HR 0.92 (95% CI: 0.77–1.11), and the difference was not statistically significant (*p* = 0.40). Patients with TPS ≥ 1% and ≥50% demonstrated OS benefits regardless of the treatment regimen. Caution is advised when using pembrolizumab in patients with NSCLC and TPS 1–49% [25]. A notable analysis by the Italian author—Alfonso Fiorelli—examined the true role of pembrolizumab in the low PD-L1 group within the KEYNOTE-042 study. In this study population, 599 patients (47%) had TPS ≥ 50%. The overall survival benefit was primarily observed in the high PD-L1 group (TPS ≥ 50%), whereas the benefit was unclear in patients with PD-L1 TPS 1–49% (mOS 13.4 vs. 12.1 months; HR 0.92, 95% CI: 0.77–1.11). These results reinforce emerging evidence that pembrolizumab monotherapy should be preferentially reserved for patients with high PD-L1 expression, whereas combination regimens incorporating chemotherapy may be more appropriate for those with intermediate PD-L1 levels, particularly in the absence of contraindications [26,27].

Smoking history emerged as a strong prognostic factor, with never-smokers exhibiting significantly poorer survival. This observation is consistent with prior studies suggesting that tobacco exposure is associated with higher tumor mutational burden (TMB), increased neoantigen load, and enhanced immunogenicity, thereby conferring greater sensitivity to immune checkpoint blockade. Conversely, tumors arising in never-smokers often harbor lower TMB and distinct molecular landscapes, which may underlie reduced responsiveness to immunotherapy [15,28].

Squamous histology was also independently associated with inferior outcomes. While immune checkpoint inhibitors have demonstrated efficacy across histological subtypes, several real-world series and meta-analyses have reported less favorable survival among patients with squamous tumors, potentially reflecting greater tumor aggressiveness, comorbidity burden, and limited availability of subsequent-line therapies [29,30]. However, our study showed an imbalance in sample size across histological subtypes, with only a small number of patients with squamous cell carcinoma. Although the result was statistically significant in the multivariate analysis, the wide confidence interval and small sample size in this subgroup substantially reduce the robustness of the conclusion. Therefore, caution is warranted when interpreting the prognostic value of histology.

### 4.3. Impact of Clinical and Molecular Factors

Although *KRAS* mutation status did not independently predict survival in our multivariate model, patients harboring *KRAS* mutations exhibited numerically longer OS (27.3 vs. 25.4 months). This trend aligns with accumulating evidence suggesting enhanced immunogenicity and improved response to immune checkpoint inhibitors among *KRAS*-mutant NSCLC, particularly in the absence of co-mutations in *STK11* or *KEAP1*. *KRAS* mutations in NSCLC are associated with a history of smoking, higher PD-L1 expression, and response to ICI monotherapy [31,32]. Exploratory retrospective biomarker analysis from the KEYNOTE-042 study to evaluate the association between high tTMB prevalence and *STK11*, *KEAP1*, and *KRAS* mutations with clinical outcomes. *KRAS* mutation patients had mOS of 28 vs. 11 months; HR 0.42; 95% CI: 0.22–0.81) compared to *KRAS* wild-type patients (mOS, 15 vs. 12 months; HR 0.86; 95% CI: 0.63–1.18). Additionally, the *KRAS G12C* mutation subgroup in first-line immunotherapy is more effective compared to patients with other *KRAS* mutations. However, some other studies indicate that there is no significant difference in the efficacy of first-line immunotherapy between *KRAS G12C* mutations and non-G12C mutations [33,34,35]. *KRAS* mutational status was available in only 42 out of 73 patients (57.5%). Moreover, only about half of these cases were analyzed using next-generation sequencing (NGS), while the remainder were evaluated using PCR-based methods. This represents an important limitation, as multiplex PCR can assess only a limited number of predefined genes and typically targets specific exons or mutation hotspots. Therefore, some patients classified as *KRAS* wild-type may actually have had undetected *KRAS* mutations. Consequently, the molecular landscape of our cohort may not have been fully characterized. In addition, given the relatively small sample size, this limitation may have further reduced the robustness of our analysis regarding the association between genomic alterations and survival outcomes. This limitation should be taken into consideration when interpreting the survival outcomes according to *KRAS* status. These limitations stem from resource constraints in our healthcare system. While EGFR-targeted therapy is covered by insurance, NGS testing and targeted drugs for other driver genes (e.g., *ALK*, *ROS1*, *KRAS*, *MET*) require out-of-pocket payment. Consequently, comprehensive genomic profiling was not performed in all patients at initial diagnosis. Nevertheless, with recent advances, more patients are now able to access NGS and novel targeted therapies to optimize treatment outcomes.

Similarly, chronic obstructive pulmonary disease (COPD) did not adversely influence survival outcomes. Recently, numerous studies have investigated the impact of COPD on NSCLC patients treated with immune checkpoint inhibitors (ICIs). Concerns have been raised that COPD may impair the anti-tumor immune response by reducing or suppressing immune cell function, and that corticosteroid use for COPD management could potentially diminish ICI efficacy. Emerging data suggest that mild-to-moderate COPD may not compromise, and may even enhance, immunotherapy responsiveness, potentially through modulation of the tumor immune microenvironment. Nonetheless, vigilant monitoring for immune-related pulmonary toxicity remains imperative in this patient population [36,37].

Furthermore, systemic inflammation plays a role in the response to ICIs among patients with advanced NSCLC. Alterations in the tumor microenvironment—characterized by high neutrophil counts and low lymphocyte infiltration—may promote angiogenesis, inhibit programmed cell death (apoptosis), and facilitate tumor growth, ultimately leading to a poorer prognosis. The LIPS-3 score is a validated prognostic classification based on the neutrophil-to-lymphocyte ratio (NLR), ECOG PS, and pre-treatment corticosteroid use. Patients were stratified by LIPS-3 score into favorable (0 risk factors, 1-year OS 78.2%), intermediate (1–2 risk factors, 1-year OS 53.8%), and poor prognosis groups (>2 risk factors, 1-year OS 10.7%) [13]. Our study shows a comparable median OS of 27.3 months in the favorable LIPS-3 group versus 19.1 months in the unfavorable group (*p* < 0.05). Although the LIPS-3 score was significantly associated with overall survival in the univariate analysis, this association did not remain statistically significant in the multivariate model after adjustment for other prognostic factors.

### 4.4. Safety Profile

Pembrolizumab monotherapy was generally well tolerated, with no new safety signals observed. Most immune-related adverse events were mild and manageable, and treatment discontinuation due to toxicity was infrequent [9,15]. The incidence of pneumonitis in our study was 8.2%, higher than reported in the KEYNOTE-024 (2.6%) and KEYNOTE-042 (3.9–5.0%) trials [9,11]. However, the majority of cases were low-grade (grade 1–2: 6.8%), with only one patient (1.4%) developing grade 3 pneumonitis that resulted in permanent discontinuation. Pooled KEYNOTE trial data showed an overall pneumonitis rate of 4.2% (grade 3–5: 1.4%) [38]. In contrast, real-world studies from Japan reported higher rates, ranging from 7.6% to 20% [20,39,40]. The relatively favorable safety profile, despite the higher incidence, may be explained by factors frequently observed in Asian real-world cohorts, including a higher prevalence of underlying chronic lung diseases (e.g., COPD), prior thoracic radiotherapy, and possible ethnic predispositions. Most events were effectively managed with corticosteroids.

Any-grade hepatotoxicity occurred in 58.9% of patients. This rate reflects all causes of liver enzyme elevation and should not be interpreted as immune-related hepatitis alone. Only one case (1.4%) of grade 3 immune-related hepatitis was observed, which resolved with corticosteroid therapy, allowing treatment resumption. Other contributing factors included chronic hepatitis B (6.8%) and liver metastases (6.8%). Notably, 81.4% of patients with elevated liver enzymes had comorbidities such as cardiovascular disease, hypertension, diabetes, or COPD requiring concomitant medications, which may have contributed to the high rate observed. The incidence of immune-related hepatitis in our study is consistent with data from KEYNOTE-024 (1.3%) and KEYNOTE-010 (0.4%) [9,41]. A pooled analysis of 36 randomized controlled trials reported severe hepatic adverse events with ICI monotherapy at 2.4% (range 0–6.1%) [42]. The reported incidence of immunotherapy-induced hepatitis varies widely, with most clinical trials reporting a low rate. However, some retrospective studies report higher rates of that adverse effect, up to 64% [43].

Anemia was noted in 46.6% of patients. This relatively high rate is likely attributable to the characteristics of our cohort, including advanced age (median 69 years), multiple comorbidities, and poor performance status, rather than pembrolizumab alone. In our cohort, no endocrinopathies were observed, and the incidences of fatigue and diarrhea were notably low (with only one reported case of diarrhea). Several factors may explain these discrepancies. First, the relatively small sample size may limit the detection of less frequent adverse events. Second, as this is a retrospective real-world study, underreporting or incomplete documentation of low-grade adverse events—particularly fatigue and mild gastrointestinal symptoms—may have occurred.

Overall, our safety data support the acceptable tolerability of pembrolizumab monotherapy in a real-world, resource-limited setting.

### 4.5. Strengths and Limitations

The major strengths of this study include its multicenter design, extended follow-up duration, and comprehensive evaluation of clinical, pathological, and molecular factors in a real-world setting. Importantly, this study provides much-needed evidence from Vietnam, where data on immunotherapy outcomes remain scarce.

Several limitations merit consideration. The retrospective design carries risks of selection bias and residual confounding. The absence of a control group prevented direct comparative effectiveness analyses. In addition, the modest sample size limited statistical power for subgroup analyses and detailed exploration of molecular interactions. Incomplete genomic profiling also restricted the assessment of important co-mutations such as STK11 and KEAP1. Furthermore, due to the retrospective design and lack of standardized iRECIST-based imaging assessment, hyperprogression and pseudoprogression could not be systematically evaluated and were not reported.

## 5. Conclusions

First-line pembrolizumab monotherapy demonstrated favorable effectiveness and acceptable safety in patients with advanced non-small cell lung cancer in real-world clinical practice in Vietnam. Improved clinical outcomes were observed among patients with high PD-L1 expression, non-squamous histology, and a history of smoking. Nevertheless, the survival benefit associated with non-squamous histology should be interpreted cautiously, given the limited number of patients with squamous histology. These findings support the use of pembrolizumab monotherapy in appropriately selected patients and provide valuable real-world evidence to guide clinical decision-making in resource-limited settings.

## Figures and Tables

**Table 1:** Baseline Characteristics of the Study Population.

Characteristic		*n* (%)
Age (years)	<70	41 (56.2)
	≥70	32 (43.8)
	Median	69 (47–92)
Sex	Male	67 (91.8)
	Female	6 (8.2)
Smoking	Never-smoker	19 (26.0)
	Current-smoker	31 (42.5)
	Former-smoker	23 (31.5)
Performance status (PS)	PS = 0	9 (12.3)
	PS = 1	46 (63.0)
	PS = 2	18 (24.7)
LIPS-3	0	29 (39.7)
	1–2	39 (53.4)
	3	5 (6.8)
Disease stage	IIIB, IIIC	5 (6.9)
	Recurrence	4 (5.5)
	IV	64 (87.7)
Brain metastasis	No	61 (83.6)
	Yes	12 (16.4)
Histological classification	Adenocarcinoma	61 (83.6)
	squamous cell carcinoma	7 (9.6)
	Other *	5 (6.8)
PD-L1 expression level	TPS 1–49%	10 (13.7)
	TPS ≥ 50%	63 (86.3)

Note: * other: not otherwise specified (NOS); invasive carcinoma: 1 patient; non-small cell carcinoma: 1 patient; carcinoma (unspecified): 1 patient; sarcomatoid carcinoma: 1 patient. LIPS-3: Lung Immuno-Oncology Prognostic Score 3. TPS: Tumor Proportion Score.

**Table 2:** Objective response rate.

Objective Response Rate	No of Patients (*n* = 73)
Complete Response (CR)	0 (0)
Partial Response (PR)	44 (60.3)
Stable Disease (SD)	14 (19.2)
Progressive Disease (PD)	15 (20.5)
Objective response rate (ORR)	44 (60.3)
Disease Control Rate (DCR)	58 (79.5)

**Table 3:** Overall survival and associated factors.

		Median OS	Univariate Analysis		Multivariate Analysis	
Factor		Months	95% CI	*p*-Value	HR (95% CI)	*p*-Value
Age (years)	<70	33.1	-	0.032	0.90 (0.38–2.10)	0.811
	≥70	24.8	5.2–44.5		1	
Sex	male	25.4	19.7–31.1	0.576	1.16 (0.27–5.04)	0.845
	female	26.1	-		1	
Performance status (PS)	ECOG 0–1	27.3	19.3–35.4	0.002	0.42 (0.14–1.26)	0.122
	ECOG 2	6.4	0.0–14.7		1	
Smoking	Never-smoker	10.7	0.9–20.5	0.001	3.14 (1.16–8.50)	0.024
	Yes	33.1	-		1	
COPD	No	26.1	16.4–35.7	0.298	0.76 (0.22–2.58)	0.658
	Yes	19.1	0.0–38.4		1	
LIPS-3	0	27.3	-	0.037	0.77 (0.28–2.17)	0.629
	1–3	19.1	6.7–31.6		1	
Histological classification	squamous cell carcinoma	13.6	4.1–23.1	0.02	4.09 (1.18–14.17)	0.026
	Non-squamous cell carcinoma	27.3	19.1–35.5		1	
Brain metastasis	No	26.1	18.1–34.1	0.192	0.60 (0.22–1.60)	0.309
	Yes	22.5	0.0–56.5		1	
PD-L1	1–49%	4.8	1.7–7.9	<0.001	3.67 (1.14–11.78)	0.029
	≥50%	27.3	18.9–35.6		1	
*KRAS*	wild-type	25.4	20.2–30.6	0.601	1.65 (0.41–6.63)	0.482
	mutation	27.3	19.6–35.0		1	

The “-” means the 95% CI is not available. OS: overall survival, HR: Hazard ratio; CI: Confidence interval, COPD: Chronic Obstructive Pulmonary Disease, LIPS-3: Lung Immuno-Oncology Prognostic Score 3.

**Table 4:** Adverse Events.

Adverse Events	Any Grade No. Patients, (%)		Grade 1–2 No. Patients, (%)		Grade 3–4 No. Patients, (%)	
Anemia	34	46.6	32	43.8	2	2.8
Neutropenia	1	1.4	0	0	1	1.4
Thrombocytopenia	4	5.5	3	4.1	1	1.4
Pneumonitis	6	8.2	5	6.8	1	1.4
Vomiting	6	8.2	6	8.2	0	0
Diarrhea	1	1.4	1	1.4	0	0
Skin reactions	11	15.1	10	13.7	1	1.4
Fever	7	9.6	7	9.6	0	0
Myositis	2	2.8	2	2.8	0	0
Hepatitis	43	58.9	37	50.7	6	8.2
Nephritis	3	4.1	3	4.1	0	0

## Data Availability

The data presented in this study are available on request from the corresponding author due to privacy and ethical restrictions (patient confidentiality).
